# Efficacy of Vitamin D supplementation in achieving an early Sputum Conversion in Smear positive Pulmonary Tuberculosis

**DOI:** 10.12669/pjms.344.14397

**Published:** 2018

**Authors:** Arbab Afzal, Rabia Rathore, Nasir Farooq Butt, Fawad Ahmad Randhawa

**Affiliations:** 1 Arbab Afzal, MD. Department of Medicine, Mayo Hospital, Lahore, Pakistan; 2 Rabia Rathore, FCPS. Department of Medicine, Mayo Hospital, Lahore, Pakistan; 3 Nasir Farooq But, FCPS. Department of Medicine, Mayo Hospital, Lahore, Pakistan; 4 Fawad Ahmad Randhawa, FCPS. Department of Medicine, Mayo Hospital, Lahore, Pakistan

**Keywords:** Pulmonary tuberculosis, Sputum conversion, Vitamin D

## Abstract

**Objective:**

To determine the efficacy of Vitamin D supplementation in achieving an early sputum conversion in vitamin D deficient smear positive pulmonary tuberculosis patients.

**Methods:**

This randomized clinical trial was done at Mayo hospital Lahore from November 2015 to August 2016. One hundred twenty patients with sputum smear positive pulmonary tuberculosis were selected and randomized to Group-A (taking anti-tuberculous therapy (ATT) only) and Group-B (taking ATT with Vitamin D supplementation). Four doses of100,000 IU of Vitamin D injection intramuscularly were given after every 14 days during intensive-phase. Sputum examination was repeated at 2^nd^, 4^th^, 6^th^, 8^th^, 10^th^ and 12^th^ weeks. Efficacy of treatment in terms of early sputum conversion between both groups was tested using Chi square and independent sample t-test was applied to compare mean values of serum vitamin D before and after treatment. P-value ≤ 0.05 was considered as significant.

**Results:**

The mean age of patients was 37.18±6.81 years in Group-A and 39.02±7.56 years in Group-B. There were 63 (52.50%) males and 57 (47.50%) females. The mean serum Vitamin D was 17.07±1.44 in Group-A and 17.23±2.37 in Group-B at baseline and at 12th week, the levels were 21.77±2.23 in Group-A and 29.24±0.72 in Group-B. In Group-A, 7 (11.7%) patients showed positive sputum examination and in Group-B, only one (1.7%) patient had positive sputum examination at 12^th^ week. The difference was statistically significant (p-value= 0.028).

**Conclusion:**

Four doses of intramuscular vitamin D given after every 14 days corrected vitamin D deficiency and improved the rate of sputum smear conversion in patients of pulmonary tuberculosis.

## INTRODUCTION

Tuberculosis (TB) is a major health problem all over the world. It is the ninth major cause of death worldwide and the leading cause from a single infectious agent, ranking above Human immunodeficiency virus infection and acquired immune deficiency syndrome (HIV/AIDS).^1^ There were an approximately 1.3 million deaths in 2016 from tuberculosis among patients who were HIV-negative and an additional 374000 deaths among HIV-positive people. An estimated 10.4 million people had Tuberculosis in 2016: 90% were adults, 65% were male, 10% were people living with HIV (74% in Africa) and 56% were in five countries: India, Indonesia, China, the Philippines and Pakistan.^1^,^2^ TB is one of the most challenging diseases for the developing countries. The spread of this disease is favored by several factors like human immunodeficiency virus (HIV)/A*cquired immune deficiency syndrome (*AIDS), low socio-economic status, overcrowding and malnutrition.^2^,^3^

The main role of vitamin D is to maintain the function of monocytes and macrophages which are linked to human innate immunity to certain infectious agents, this role is very important in body’s natural defense against infection in which macrophages plays a vital role in pathogenesis. Vitamin D act by combining the nuclei receptor on affected cells so both abnormality in receptor function and structure or low vitamin D level alter the immunity against tubercle bacillus.^4^

Almost 50% of the world’s population is affected by vitamin D deficiency.^5^ Deficiency of vitamin D commonly occurs because of decrease exposure of ultraviolet radiation due to reduced sun exposure that eventually resulted in lesser vitamin D production in skin.^6^ Low serum vitamin D levels are associated with higher risk of active tuberculosis.^4^ Sato S et al., reported that the deficiency of vitamin D is 87% in pulmonary tuberculosis patients.^5^

The goal of treatment for TB is to eradicate mycobacterium tuberculosis (MTB) using combination of drugs. Maintaining adequate levels of Vitamin D during the treatment of TB helps in achieving this goal.^6^ A recent study by Martineau AR et al has reported that administration of four doses of 1,00,000 IU of vitamin D-3 increased vitamin D blood levels in patients taking intensive-phase treatment for pulmonary TB and reduction in the median time to sputum conversion by at least seven days.^7^

TB is prevalent in our country and resistance against anti-tuberculous drugs is also very high. Hence, there is a need to find out the beneficial effects of vitamin D supplementation in our population in achieving an early sputum conversion in smear positive pulmonary tuberculosis. This will help to establish the efficacy of vitamin D supplementation in achieving sputum conversion and it will provide adequate evidence which can be utilized in coming future to manage pulmonary tuberculosis in a much better way and achieving sputum conversion. The purpose of using four doses of Vitamin D in this study was the higher prevalence of vitamin D deficiency in our population as compared to the western world.^7^ Only few local studies are available on use of Vitamin D in the management of Pulmonary TB, that too with limited sample size. This study will help us to establish the bench mark in the better management of pulmonary TB in near future which will be cost effective and beneficial for the patients.

## METHODS

It was a Randomized clinical trial conducted at West Medical ward, Institute of Chest Medicine and PMRC Center of Mayo hospital Lahore from November 2015 to August 2016 with clinical trial number research registry 3342 registered at www.researchregistry.com. Sample size of 120 patients (60 patients in each group) using non-probability purposive sampling was estimated by using 90% confidence level, 9% absolute precision with expected percentage vitamin D as 100% and placebo group as 76.7%.

Patients aged above 18 years, newly diagnosed with pulmonary tuberculosis (as determined by symptoms and signs, a positive acid-fast bacilli (AFB) sputum smear and Chest X-ray findings) were included in study.

Patients with extrapulmonary tuberculosis or multi-drug-resistant tuberculosis (MDR-TB), pregnant or lactating females, serum calcium above 10.5 mg/dL, past history of renal stones, liver and/or renal disorder, patients on or had anti-tuberculous therapy, steroids, antiepileptics, cytotoxic or immunosuppressive drugs were excluded.

Ethical approval was obtained from Institutional Review Board. After taking informed consent, 120 patients were selected and randomized to either Group-A (not receiving Vitamin D) or treatment Group-B (receiving Vitamin D) using lottery method. Patients were diagnosed for pulmonary tuberculosis on sputum analysis for Acid- Fast Bacillus (AFB) with at least 1+ve sputum in two out of three consecutive samples or 2+ve sputum in at least one out of three samples. Early Sputum conversion was the conversion of patient’s sputum for AFB to negative in three consecutive day samples done four weeks after initiation of treatment. Patients were labelled as Vitamin D deficient with serum vitamin D levels below 20 ng/ml. Demographic information (like name, age, sex, height and weight) were also obtained. Complete blood count, fasting blood sugar, liver function tests, serum creatinine, serum calcium and serum Vitamin D levels were done at baseline. Group-A, patients were treated with weight based anti-tuberculous combination therapy (isoniazid (INH), rifampicin, pyrazinamide and ethambutol) once a day before breakfast daily for three months (Intensive Phase) then with INH 300mg and rifampicin 600mg (once a day) for next six months (Continuation phase). In Group-B, patients were treated with weight based anti-tuberculous combination therapy (isoniazid, rifampicin, pyrazinamide and ethambutol) once a day before breakfast daily for three months (Intensive Phase) along with four doses of 100,000 IU of vitamin D intramuscularly every fortnightly then with INH 300mg and rifampicin 600 mg (once a day) for next six months (continuation phase). Sputum examination was done at two, four, six, eight, 10 and 12 weeks follow up for all patients. Complete blood count, fasting blood sugar, liver function tests and serum creatinine were also done at two, four, six, eight, 10 and 12 weeks. Patients with transaminitis and elevated serum creatinine were dropped out of the study. At the end of 3^rd^ month, serum calcium and serum vitamin D levels were done. All this information was recorded on predesigned proforma.

Data was entered in SPSS 23. Quantitative variables like age was presented as mean ±S.D. Quantitative variables were presented as frequency and percentages. Efficacy of treatment in terms of early sputum conversion between both groups was tested using Chi square and independent sample t-test was applied to compare mean values of serum vitamin D before and after treatment amongst both groups. P-value ≤ 0.05 was considered as significant.

## RESULTS

The mean age of patients in Group-A was 37.18±6.82 years and in Group-B were 39.02±7.56 years. The mean ages were statistically same among the two groups (p-value= 0.166). There were 63 (52.50%) males and 57 (47.50%) females. The mean Body mass index (BMI) of patients in Group-A was 23.1092±3.247 kg/m^2^ and in Group-B was 23.684±2.7559 kg/m^2^ which was statistically same in two study groups (p-value=0.298). Table-I compares the laboratory investigations in both groups at different visit. Table-II shows the comparison of mean Vitamin D levels in both groups and Table-III elaborates the comparison of sputum conversion amongst patients in both groups at 12^th^ week. Three patients in Group-A and two patients in Group-B dropped out due to Anti-tuberculous therapy induced hepatitis. Similar number of new patients, who fulfilled the inclusion criteria, were enrolled in respective groups and passed through the same protocol. No patient was lost during follow up. All patients completed the treatment.

**Table-I:** Comparison of laboratory Investigations in both groups at different visits.

Laboratory Investigations		Study groups	Mean	SD	p-value
Hemoglobin (gm/dl)	At 1st visit (Baseline)	Group-A	12.92	1.04	<0.001
		Group-B	11.69	1.35	
	At 12th week	Group-A	14.26	0.75	0.154
		Group-B	14.08	0.63	
White Blood Cells (cells/mm3)	At 1st visit (Baseline)	Group-A	9.5983	1.93509	<0.001
		Group-B	7.4283	2.56434	
	At 12th week	Group-A	5.6267	0.36354	0.683
		Group-B	5.6000	0.34933	
Serum calcium (mg/dl)	At 1st visit (Baseline)	Group-A	9.01	0.39	<0.001
		Group-B	8.80	0.22	
	At 12th week	Group-A	9.43	0.11	0.182
		Group-B	9.40	0.14	

**Table-II:** Comparison of mean Vitamin D levels in both groups.

Vitamin D levels (ng/ml)	Study groups	Mean	S.D	p-value
1st visit (Baseline)	Group-A	17.07	1.44	0.056
	Group-B	17.23	2.37	
2^nd^ Visit (12thweek)	Group-A	21.77	2.23	<0.001
	Group-B	29.24	0.72	

**Table-III:** Comparison of Sputum conversion at last visit.

		Study groups		Total
		Group-A	Group-B	
Sputum Examination at 12th week	Positive	7	1	8
		11.7%	1.7%	6.7%
	Negative	53	59	112
		88.3%	98.3%	93.3%
Total	60	60	120	
	100.0%	100.0%	100.0%	

p = 0.028

## DISCUSSION

We did this study to assess the role of supplementation of Vitamin D in treatment of patients suffering from pulmonary tuberculosis (PTB). The risk factors for acquiring PTB are poor nutritional and socioeconomic status, houses having inadequate sunlight, bad sanitary conditions, malnutrition, illiteracy and low BMI.^8^-^10^ All these predisposing factors increase the likelihood for acquiring tuberculosis by reducing hemoglobin levels in addition to developing other serious diseases.^11^,^12^ Studies have shown the momentous relation between deficiency of vitamin D and PTB.^13^-^15^ Our study also showed similar evidence as the mean value of Vitamin D at baseline was 17.07 ng/ml and 17.23 ng/ml in Group A and B respectively which was well below the reference range, indicating vitamin D deficiency. This has also been detected in different studies that first line anti-tuberculous therapy (ATT) drugs especially isoniazid and rifampicin influence Vitamin D metabolism causing low levels of vitamin D during course of the treatment.^5^,^16^ These low levels of Vitamin D induced by ATT might even slightly increase the serum calcium levels during the treatment regardless of the regulation of calcium levels by Vitamin D.^17^,^18^ Nursyam and colleagues found a notable improvement in the rate of microscopic AFB sputum conversion with supplementation of 0.25 mg vitamin D daily during intensive phase of anti-tuberculous treatment.^11^ Kota et al.^19^ established that addition of Vitamin D (60,000 IU per week) and Calcium carbonate one gram daily with standard treatment of Pulmonary TB decreased the time to microscopic sputum smear conversion. Vitamin D act by enhancing the host immune response to mycobacterium tuberculosis by suppressing interferon gamma and hence reduce inflammation. Salahuddin and associates^20^ did a study on 259 subjects to check the influence of Vitamin D supplementation on pulmonary Tuberculosis. They used six lac IU of intramuscular vitamin D3 for two doses and assessment was done at four, eight and 12 weeks. Result showed that patients receiving vitamin D demonstrated significantly greater mean weight gain and lesser residual disease by chest X-ray. They concluded that supplementation with vitamin D enhanced clinical, radiographic improvement and increased host immune activation; thus, suggesting a definite role for vitamin D in the management of pulmonary TB. Our study also showed that in treatment Group-B, 59 (98.3%) patients had sputum clearance at 12 weeks as compared to 53 (88.3 %) patients in Group-A, who were not taking Vitamin D.

Few studies have shown that there is no proven role of Vitamin D in rate of sputum conversion in patients on treatment for pulmonary TB.^21^,^22^ Ganmaa D et al. did a study including 390 patients with Pulmonary TB to see the effects of adjunctive vitamin D supplementation on the outcome of sputum culture conversion. Their findings suggested that high-dose vitamin D did not improve the rate of sputum conversion.^23^ Azam F et al.^18^ did a study on 86 patients to assess the role of adjuvant Vitamin D on pulmonary TB with respect to disease severity. They used 0.6 million units of Vitamin D intramuscular at baseline and repeat the same dosage at 6th week of the study along with standard ATT. Laboratory investigations were done at baseline and at 75th day of therapy. Microscopic examination of sputum was done fortnightly till sputum conversion. Results showed that statistically significant changes were seen in the mean number of days required for microscopic Acid-Fast Bacilli sputum conversion in the Vitamin D treatment and placebo groups which were 49 days and 61 days respectively (p=0.032). It was concluded that adjuvant Vitamin D played a noteworthy role in the management of pulmonary TB as evident by early sputum conversion by 12 days in comparison to placebo group. In our study, rather than checking the total number of days required for sputum conversion, we checked the total number of patients with sputum smear conversions at fixed number of days i.e. at 12 weeks. The results of our study showed that in Group-A (not received Vitamin D), 7 (11.7%) patients showed a positive sputum examination and in Group-B (who received Vitamin D), only one (1.7%) patient had positive sputum examination at 12^th^ week. Statistically significant sputum conversion results were observed in group-B as compared to group-A (p-value= 0.028).

Vitamin D supplementation increases the calcidiol levels in blood and thus has an important role in the prevention and management of infections including pulmonary TB. Coussens AK et al.^24^ did a clinical trial on 95 patients of Pulmonary Tuberculosis to see the immunomodulatory effects of administering vitamin D. Results showed that addition of Vitamin D hastens microscopic AFB sputum smear conversion and improved treatment-induced resolution of lymphopenia, monocytosis, hypercytokinaemia, and hyperchemokinaemia. It was concluded that addition of vitamin D supplementation had a significant role in the management of pulmonary TB by accelerating resolution of inflammatory responses associated with increased risk of mortality. Therefore, in accordance with findings and above-mentioned studies, it is suggested that high doses of vitamin D supplementation improve the rate of sputum smear conversion in pulmonary TB patients and also enhance the host immune response in patients who are vitamin D deficient. Nonetheless, only a small number of studies show considerable change in sputum assessment, counting ours. Further studies should be encouraged in different domains of this very aspect.

The study results can be generalized to the population of sputum smear positive patients having vitamin D deficiency who are treatment naïve.

### Limitations

Our study has shown better outcomes in sputum smear conversion with supplementation of vitamin D in Pulmonary TB patients, but it has some limitations. Firstly, it is not a multicenter trial and sample size was limited depending upon the study design and prerequisites. Secondly, Placebo was not given to Group-A so the difference could also be attributed to the psychological effect of intramuscular injection of Vitamin D in the intervention group. Moreover, the study only included treatment naïve patients, the study results cannot be generalized to patients with reactivation of the disease or patients with disseminated tuberculosis. Further interventional studies are needed which should try to answer all these conflicting conclusions.

## CONCLUSION

Vitamin D supplementation in vitamin D deficient sputum smear positive Pulmonary Tuberculosis patients improved the rate of sputum smear conversion. The researchers recommend supplementing vitamin D to smear positive pulmonary tuberculous patients who are vitamin D deficient.

### Authors’ Contribution

**AA** conceived and designed the study.

**RR and AA** did data collection and manuscript writing.

**NFB and RR** did statistical analysis & editing of manuscript.

**FAR** did review and final approval of manuscript.
